# Potential value of the calibrated automated thrombogram in patients after a cerebral venous sinus thrombosis; an exploratory study

**DOI:** 10.1186/s12959-021-00335-1

**Published:** 2021-11-04

**Authors:** Myrthe M. van der Bruggen, Bram Kremers, Rene van Oerle, Robert J. van Oostenbrugge, Hugo ten Cate

**Affiliations:** 1 Department of Internal Medicine, Maastricht University Medical Centre, Maastricht, the Netherlands; 2 Department of Biochemistry, Cardiovascular Research Institute Maastricht, Maastricht, the Netherlands; 3 Clinical Diagnostic Laboratory, Maastricht University Medical Center, Maastricht, the Netherlands; 4 Department of Neurology, Maastricht University Medical Centre, Maastricht, the Netherlands

## Abstract

**Background:**

Cerebral venous sinus thrombosis (CVST) is a relatively rare, but potentially lethal condition. In approximately 15% of the patients, the cause of CVST remains unclear. Conventional clotting tests such as prothrombin time and activated partial thromboplastin time are not sensitive enough to detect prothrombotic conditions nor mild haemostatic abnormalities. The calibrated automated thrombogram (CAT) is a physiological function test that might be able to detect minor aberrations in haemostasis. Therefore, we aimed to detect the presence of a prothrombotic state in patients who endured idiopathic CVST with the CAT assay.

**Methods:**

*Five adult patients* with an idiopathic, radiologically proven CVST that had been admitted during the past 3 years were included in this study. The control group consisted of *five* age/gender matched healthy volunteers. Exclusion criteria were known haematological disorders, malignancy (current/past) or hormonal and anticoagulant therapy recipients. We obtained venous blood samples from all participants following cessation of anticoagulation. Using the CAT assay, we determined lag time, normalized endogenous thrombin potential (ETP), ETP reduction and normalized peak height. In addition, prothrombin concentrations were determined.

**Results:**

We found no significant differences in lag time (4.7 min [4.5–4.9] vs 5.3 min [3.7–5.7], *p =* 0.691), normalized ETP (142% [124–148] vs 124% [88–138], *p* = 0.222), ETP reduction (29% [26–35] vs 28% [24–58], *p* > 0.999), and normalized peak height (155% [153–175] vs 137 [94–154], *p* = 0.056) between patients and their age/gender matched controls. In addition, prothrombin concentrations did not significantly differ between patients and controls (120% [105–132] vs 127% [87–139], *p* > 0.999).

**Conclusion:**

Reasons for absent overt hypercoagulability within this study population may be the small patient sample, long time since the event (e.g. 3 years) and avoidance of acquired risk factors like oral contraception. Given the fact that CVST is a serious condition with a more than negligible risk of venous thrombosis event recurrence, exclusion of clinically relevant hypercoagulability remains a challenging topic to further study at the acute and later time points, particularly in patients with idiopathic CVST.

## Introduction

Cerebral venous sinus thrombosis (CVST) is a relatively rare subtype of stroke. The incidence of this disease reported in the literature is approximately 3 per 100.000 per year, affecting more women than men [1, 2]. Because of its rare occurrence the incidence of this disease might be underestimated, due to unawareness of its diagnosis. In addition, CVST is difficult to diagnose as it has a variable clinical presentation and may be challenging to confirm radiologically [3]. Complications include subarachnoid haemorrhage, cranial nerve palsy, epilepsy and transient ischemic attacks [4–6]. Taken together, the consequences of CVST can be severely incapacitating, and potentially life threatening [4]. This is illustrated by a case from a 19-year old female who visited the emergency room with acute headache in the last 24 h. The pain was progressive, the patient experienced nausea and vertigo, and she had vomited several times. In addition, she was both phono- and photophobic. Her medical history did not show any peculiarities and the family history was negative for thrombotic diseases. She did not suffer head trauma or infections, nor did she use any hormonal/contraceptive therapy. Magnetic resonance imaging showed a thrombosis of the jugular vein, sigmoid sinus, right transverse sinus and the distal part of the sagittal sinus. Thrombophilia analysis did not show any of the common traits including factor V Leiden, prothrombin 20210 or inhibitor deficiencies. Despite adequate treatment with anticoagulants, the patient still regularly experienced loss of vision and headache years after the event. This case illustrates how severe CVST can influence a (young) patient’s life.

Recent occurrences of CVST in the setting of Covid-19 vaccination, triggered interest for this disorder [7]. There are several risk factors that contribute to the development of CVST. Examples are prothrombotic conditions such as protein C -, protein S -, and antithrombin deficiency. Furthermore, tumours, haematological disorders and the use of oral hormonal contraception are associated with CVST [1, 8, 9]. The risk factors that contribute to the development of CVST can be identified in most patients. However, in approximately 15–20% of patients no cause or risk factor is identified [1, 10]. Subsequently, monitoring this patient group and estimating the risk for recurrence is rather difficult [11].

In general, hypercoagulability is a key element in venous thrombosis and in addition to the mentioned thrombophilic traits, acquired factors like oral contraceptives have an important impact on coagulation through an acquired resistance against activated protein C [12]. The intrinsic coagulation properties can be assessed through the calibrated automated thrombogram (CAT). The CAT, developed by Hemker and coworkers, is a semi-automated thrombin generation technique, which provides the ability to monitor thrombin concentrations in time, as the substrate for thrombin is fluorescently labeled [13, 14]. The CAT method is currently well-accepted as a research tool and has proven to be useful in several different domains such as platelet-plasma interactions, detection and quantification of thrombotic/bleeding tendency, and control of pro-coagulant and antithrombotic therapy [12, 15–17]. During a curiosity driven exploration of the CAT data in consecutive patients referred to the vascular outpatient clinic, we observed three patients who suffered from idiopathic CVST and had substantial elevations in thrombin generation (i.e. mean endogenous thrombin potential (ETP) 190%, mean normalized peak 352%) without any reasonable explanation.

Based on this unpublished observation we designed the present study to investigate whether abnormal CAT responses would indeed be a consistent finding in patients who suffered a CVST.

## Methods

Adult patients who endured CVST without known cause or risk factors were included in this patient study. We searched hospital records for CVST patients between January 1st 2012 and May 24th 2017 using the Dutch financial coding system for hospital care (DBC-codes). There is no specific code for CVST or for cerebral venous thrombosis. Therefore, all records of patients assigned to the code “ischemic stroke”, “haemorrhagic stroke”, “headache” and “not other specified” were screened. Based on this search strategy we identified 29 CVST patients. Next, patients under 18 years of age, patients with known coagulation disorders, malignancy (in the past), using hormonal contraception or other hormonal therapy, or anticoagulants and patients who are mentally disabled, were excluded from this study.

Blood plasma from healthy age and gender matched volunteers was used as reference material. Healthy volunteers were recruited through advertisements at the faculty of health, medicine and life sciences at Maastricht University. The same exclusion criteria as with patients applied.

All subjects who were eligible for inclusion in this study underwent a venepuncture and filled in a questionnaire with regard to thrombotic risk factors. This was done to place outcome measures in a clinical perspective.

All subjects were informed and provided written consent.

The study was performed at Maastricht University Medical Centre (MUMC+) and approved by the local medical ethical committee (Medical ethical committee MUMC, approval number; NL 63775.068.17).

### Blood collection and storage

Four 9 mL tubes with 3.2% trisodium citrate were collected through antecubital venipuncture. The blood was processed to platelet poor plasma (PPP) within 1 h after collection via previously described methods [18, 19]. PPP was stored at -80 °C until analysis [20]. All samples were analyzed at one time point to prevent repeated freeze-thaw cycles. Plasma from healthy volunteers was processed and stored in the same manner, with the same number of freeze-thaw cycles as plasma from patients.

### Markers of coagulation

#### Thrombin generation

The coagulation potential in plasma was assessed using the CAT assay (Thrombinoscope BV, Maastricht, the Netherlands). Within this method, low-affinity fluorogenic substrate for thrombin (Z-Gly-Gly-Arg-AMC; Bachem, Bubendorf, Switzerland) is added to allow continuous monitoring of thrombin formation. For each measurement, 80 μL of human PPP was added to 20 μL of fluorogenic substrate, 20 μL of trigger reagent and calcium chloride, as previous reported [19, 21]. The CAT assay was performed with and without the presence of soluble thrombomodulin (TM; Asahi Kasei Pharma Corporation, Tagata, Japan), to enable protein C depend testing [12]. TG curves were calculated using Thrombinoscope software (Thrombinoscope, Maastricht, The Netherlands). Analysis resulted in four main outcome parameters: 1. Lag time; the time until clotting occurs. 2. ETP; the total amount of thrombin formed during the measurement, i.e. the area under the curve. 3. Peak height; maximum amount of thrombin generation. 4. Peak reduction; time needed for clot degradation [19].

#### Prothrombin levels

Being an important determinant of the CAT, prothrombin was measured with a one stage FII assay on a Siemens BCSxp instrument according to the manufacturer’s instructions.

### Statistical analysis

Baseline characteristics were collected and tabulated. Differences in thrombin generation outcome measures between patients and controls were analyzed using the Mann-Whitney U test (nonparametric) because of the small sample size. Results are shown as the median and 25th–75th percentile.

*P*-values < 0.05 were considered statistically significant. All analyses were performed using GraphPad Prism version 7 for Windows, GraphPad Software, La Jolla California USA, www.graphpad.com.

## Results

### Baseline characteristics

**Fig. 1:**
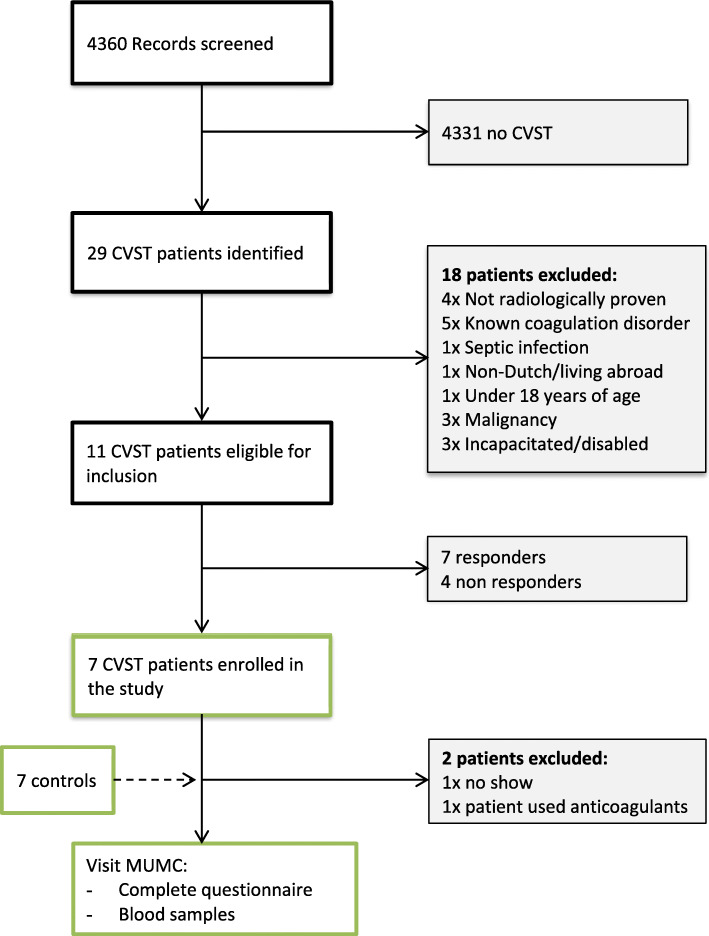
Flowchart inclusion. CVST = Cerebral venous sinus thrombosis. MUMC = Maastricht University Medical Centre

Eleven patients were eligible for participation in the study. Four patients were not interested in participating. From the remaining seven patients, one patient did not appear at the appointment and one patient used anticoagulant drugs despite the screening efforts. Consequently, 5 patients and 5 controls were enrolled in the study. A flow-chart of the inclusion- and exclusion process can be found in Fig. 1.

At the time of the event, there was no relevant medical history or use of medication that was expected to provoke CVST. Medical history of the five patients at the time of the event included: polyarticular juvenile idiopathic arthritis (1 patient), exertional headaches (1 patient), and hypertension (1 patient). Medication use included TNF-α blocker (1 patient), metoprolol (1 patient), ibuprofen and paracetamol (1 patient). Patient characteristics of all study participants at the time of inclusion are shown in Table 1. No differences in key risk factors and relevant medication between patients and controls were observed.

**Table 1:** Baseline characteristics

	Patients	Controls
Female gender (%)	100%	100%
Age in years (mean)	42 (±12)	46 (±13)
Years after event (mean)	2,5	
Antiplatelet medication	0	0
Anti-inflammatory drugs	0	1
Alcohol consumption (U/week)	1	2
Smoking	0	0

### Markers of coagulation

#### 1.1 No significant differences in thrombin generation were observed

**Fig. 2:**
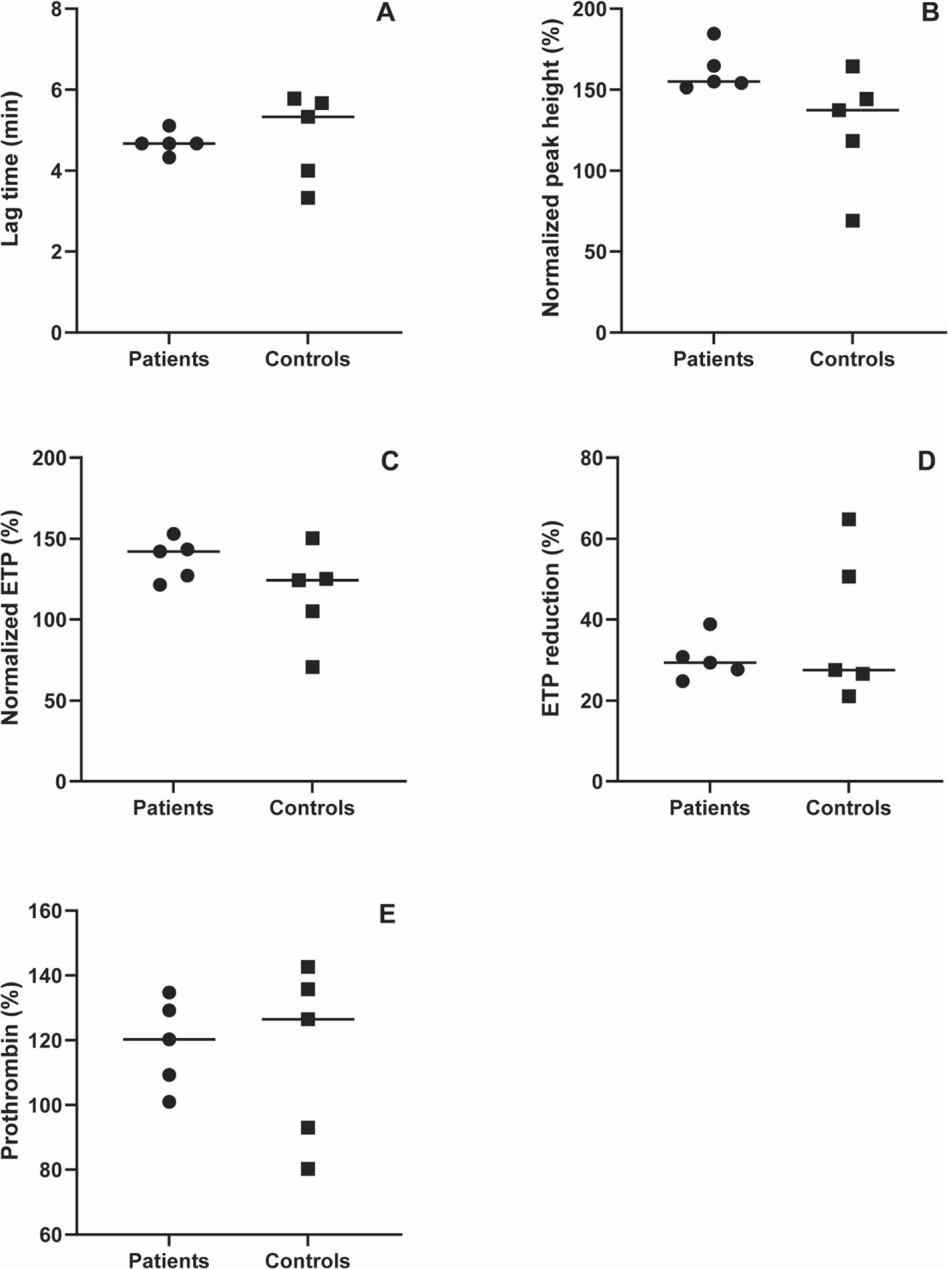
Thrombin generation and prothrombin measurements in cerebral venous sinus thrombosis (CVST) patients and healthy controls. (**A**) Lag time (**B**) Normalized peak height. (**C**) Normalized endogenous thrombin potential (ETP). (**D**) ETP reduction (**E**) Prothrombin concentration

The CAT thrombin generation assay was performed and lag time, ETP, ETP reduction and peak height were assessed [Table 2]. There was no significant difference in lag time between patients and controls (*p* = 0.691) [Fig. 2A]. Although normalized peak height and ETP were both higher in patients than controls, neither difference was statistically significant (*p* = 0.056, *p* = 0.222 respectively) (Table 2 and Fig. 2B/C). ETP reduction, which is the difference in ETP determined with and without presence of TM, was similar between the groups [Table 2 and Fig. 2D].

**Table 2:** Thrombin generation outcome measures shown as median [25–75 percentiles]. No significant differences were observed. ETP = endogenous thrombin potential

	Patients	Controls	*P*-value
Lag time (min)	4.7 [4.5–4.9]	5.3 [3.7–5.7]	0.691
Normalized ETP (%)	142 [124–148]	124 [88–138]	0.222
ETP reduction (%)	29 [26–35]	28 [24–58]	> 0.999
Normalized Peak Height (%)	155 [153–175]	137.4 [94–154]	0.056
Prothrombin concentration (%)	120 [105–132]	127 [87–139]	> 0.999

#### 1.2 No significant differences in prothrombin concentration were observed

Prothrombin was tested as it is known to be a main determinant of peak height and ETP [22–24]. No significant difference in prothrombin concentration was observed [Table 2/Fig. 2E].

## Discussion

The present study assessed the presence of unexplained hypercoagulability in patients well beyond the acute phase of a CVST. We analysed thrombin generation by CAT based on a previous finding of elevated TG levels in 3 patients with CVST seen at the outpatient clinic. Two of the original 3 patients were included in the present study, the third patient was on rivaroxaban and could for that reason not be studied.

Thrombin generation via the CAT method was performed in order to assess hypercoagulability. The first outcome parameter we considered was lag time. As expected, lag time did not significantly differ between patients and controls, as idiopathic hypercoagulability is mainly reflected by an increase in ETP and peak height [23, 25].

Subsequently we determined ETP, ETP reduction and peak height. ETP did not significantly differ between patients and controls. We tested ETP reduction by adding TM. Binding of thrombin to TM activates Protein C, a potent anticoagulant factor [13, 26]. We did not observe a significant difference in ETP reduction.

In addition, peak height of the thrombin generation curve was determined. Although there is a visual difference between the two groups (Fig. 2B), this did not reach statistical significance, probably due to the small sample size.

Based on a previous study which showed increased ETP and peak height in patients after deep venous thrombosis up till 2 years after the event [24], we expected to detect a similar difference in our study population. Other studies do support the findings of increased ETP and peak height after a thrombotic event [24]. The clinical relevance would be found in the ability to predict recurrent thrombotic events. However, results regarding the predictive value of the thrombin generation assay on development of a secondary event (venous thrombotic event) vary widely [20, 27–31]. It should be mentioned that some studies used whole blood, others used PPP or platelet rich plasma, which makes it difficult to compare the outcomes. Nevertheless, we can conclude that the role of thrombin generation as a predictor is still uncertain.

Patients with a first episode of CVST, a potentially devastating condition, are usually treated with anticoagulation for a limited time period, ranging from 3 to 6 months. This time is defined, considering the risk of recurrent disease after cessation of anticoagulation as low. However, this risk is not negligible and incidences ranging from 3 to 18% – depending on the risk factors – for development of recurrence CVST and/or other venous thrombotic events (VTE) have been reported [1, 10, 11]. Literature suggests that the risk of recurrence is highest up till one till 2 year(s) after the event [11, 32]. Therefore, characterizing thrombosis risk after stopping anticoagulation may be useful.

In all patients with CVST, avoidable risk factors like oral contraceptives are typically recorded and eliminated, whereas in younger individual’s variable thrombophilia screening is done. Since thrombin generation might have been a convenient single test for detecting thrombophilia, we focused on its use in this population.

### Study limitations

Limitations of this study include the small number of participants remaining after an extensive search and selection process. CVST is a relatively rare disease and we selected an even smaller group by only including idiopathic CVST patients. The direction of some of the TG comparisons showed a trend towards hypercoagulability in the patients and it may be assumed that in a larger population true differences may emerge. Furthermore, we excluded patients with a severe course of the disease, as we excluded disabled/incapacitated subjects. This might have led to selection bias in the current population. Finally, the time after the event might be too long to be able to detect hypercoagulability and in case of risk factors like oral contraceptives, these were eliminated after the first event. We included patients after 3 years on average. It might simply be that the hypercoagulable state tends to normalize after this time interval.

## Conclusions

In this pilot study we did not find any significant aberration in haemostasis between patients that suffered from CVST in the past, and age/gender machted controls. This is potentially due to a small sample size and relatively long follow-up time. Given the fact that CVST is a serious condition with a more than negligible risk of (VTE) recurrence, exclusion of clinically relevant hypercoagulability remains a challenging topic to further study at the acute and later time points, particularly in patients with idiopathic CVST.

## Data Availability

The datasets used and/or analysed during the current study are available from the corresponding author on reasonable request.

## Abbreviations

CAT: Calibrated automated thrombogram
CVST: Cerebral venous sinus thrombosis
DBC: Diagnosis-treatment combinations
ETP: Endogenous thrombin potential
MUMC: Maastricht university medical centre
PPP: Platelet poor plasma
TM: Thrombomodulin
VTE: Venous thrombotic event
